# FORWARD WITH FORTITUDE: THE VIRGINIA TECH CENTER FOR GERONTOLOGY

**DOI:** 10.1093/geroni/igae098.0839

**Published:** 2024-12-31

**Authors:** Carlisle Shealy, Pamela Teaster

**Affiliations:** Virginia Tech Center for Gerontology, Blacksburg, Virginia, United States; Virginia Tech., BLACKSBURG, Virginia, United States

## Abstract

The Center for Gerontology at Virginia Tech (VTCfG) celebrated its 45th year of existence and annual celebration of gerontology. The VTCfG offers two graduate certificates—the Graduate Certificate in Gerontology (for seated students) and the Certificate for Leadership in an Aging Society (for online students). The VTCfG has five Core Faculty Gerontologists, 95 Faculty Affiliates, one International Faculty Affiliate, 12 Center Associates, 140 Graduate Certificate Recipients, and over 10 graduate students pursuing the Graduate Certificate in Gerontology at any one time. The VTCfG has a Futures Board of 18 members from various entities and organizations in the state and nationally and participates in approximately 20 funded research projects at one time. Drs. Shealy and Teaster will provide a history of the VTCfG, challenges faced through the years, and opportunities and directions for the next 50 years.

